# Sustained Calcium(II)-Release to Impart Bioactivity in Hybrid Glass Scaffolds for Bone Tissue Engineering

**DOI:** 10.3390/pharmaceutics12121192

**Published:** 2020-12-08

**Authors:** Dzmitry Kuzmenka, Claudia Sewohl, Andreas König, Tobias Flath, Sebastian Hahnel, Fritz Peter Schulze, Michael C. Hacker, Michaela Schulz-Siegmund

**Affiliations:** 1 Pharmaceutical Technology, Institute of Pharmacy, Faculty of Medicine, Leipzig University, 04317 Leipzig, Germany; dzmitry.kuzmenka@uni-leipzig.de (D.K.); claudia_sewohl@gmx.de (C.S.); mhacker@uni-leipzig.de (M.C.H.); 2 Department of Prosthetic Dentistry and Dental Materials Science, Leipzig University, 04103 Leipzig, Germany; andreas.koenig@medizin.uni-leipzig.de (A.K.); sebastian.hahnel@medizin.uni-leipzig.de (S.H.); 3 Department of Mechanical and Energy Engineering, University of Applied Sciences Leipzig, 04277 Leipzig, Germany; tobias.flath@htwk-leipzig.de (T.F.); peter.schulze@htwk-leipzig.de (F.P.S.); 4 Institute of Pharmaceutics and Biopharmaceutics, Heinrich Heine University Duesseldorf, 40225 Duesseldorf, Germany

**Keywords:** Calcium(II) release, bioactivity, sol-gel, hybrid glass scaffolds, bone tissue engineering

## Abstract

In this study, we integrated different calcium sources into sol-gel hybrid glass scaffolds with the aim of producing implants with long-lasting calcium release while maintaining mechanical strength of the implant. Calcium(II)-release was used to introduce bioactivity to the material and eventually support implant integration into a bone tissue defect. Tetraethyl orthosilicate (TEOS) derived silica sols were cross-linked with an ethoxysilylated 4-armed macromer, pentaerythritol ethoxylate and processed into macroporous scaffolds with defined pore structure by indirect rapid prototyping. Triethyl phosphate (TEP) was shown to function as silica sol solvent. In a first approach, we investigated the integration of 1 to 10% CaCl_2_ in order to test the hypothesis that small CaCl_2_ amounts can be physically entrapped and slowly released from hybrid glass scaffolds. With 5 and 10% CaCl_2_ we observed an extensive burst release, whereas slightly improved release profiles were found for lower Calcium(II) contents. In contrast, introduction of melt-derived bioactive 45S5 glass microparticles (BG-MP) into the hybrid glass scaffolds as another Calcium(II) source led to an approximately linear release of Calcium(II) in Tris(hydroxymethyl)aminomethane (TRIS) buffer over 12 weeks. pH increase caused by BG-MP could be controlled by their amount integrated into the scaffolds. Compression strength remained unchanged compared to scaffolds without BG-MP. In cell culture medium as well as in simulated body fluid, we observed a rapid formation of a carbonated hydroxyapatite layer on BG-MP containing scaffolds. However, this mineral layer consumed the released Calcium(II) ions and prevented an additional increase in Calcium(II) concentration in the cell culture medium. Cell culture studies on the different scaffolds with osteoblast-like SaOS-2 cells as well as bone marrow derived mesenchymal stem cells (hMSC) did not show any advantages concerning osteogenic differentiation due to the integration of BG-MP into the scaffolds. Nonetheless, via the formation of a hydroxyapatite layer and the ability to control the pH increase, we speculate that implant integration in vivo and bone regeneration may benefit from this concept.

## 1. Introduction

Mechanically stable macroporous 3D printed hybrid glass scaffolds with a well-defined architecture and bioactive characteristics appear ideal for an application in the field of bone regeneration. Hybrid glass scaffolds consisting of organic and inorganic components dispersed at the nano-meter scale have been shown to offer good mechanical stability and elastic behaviour upon compression, biodegradability with non-toxic degradation products, bioactivity, excellent scaffold textural properties and open porous structure for cell invasion and proliferation [1,2,3,4]. Incubation of bioactive glasses in simulated body fluid (SBF) leads to a specific chemical response with a mineral deposition on its surface, supporting a strong bonding to the surrounding tissue [4,5,6,7,8,9]. Glass bioactivity is substantially enhanced by addition of Calcium(II) ions [10]. For the incorporation of inorganic calcium sources (CaCl_2_, Ca(NO_3_)_2_) [11,12], a thermal treatment over 400 °C is generally required, whereas network incorporation does not take place at room temperature [13]. 400 °C, however, are incompatible with organic polymers used for the hybrid glass preparation [14]. The missing integration of Calcium(II) in the silica network results in the recrystallization of CaCl_2_ on the material’s surface upon gel drying and causes wash out, with a burst release when dried materials are incubated in an aqueous medium [15,16,17,18]. Subsequently, significant changes in the implant microenvironment, including increased ion concentration and related cytotoxicity, may occur [19,20,21,22]. Alternatively, organic Calcium(II) precursors hydrolysing at ambient conditions may be used. For instance, recently, the concept of successful Calcium(II) incorporation using calcium ethoxide was proved by Lao [23], Bossard [24] and Granel [25]. Poologasundarampillai [26,27] and Li [28] successfully applied calcium methoxyethoxide. However, the moisture sensitivity and supplier-dependent variability of these precursors may change the sol gelation time significantly constraining their application [13]. Furthermore, the possible toxicity of these precursors should be considered [29]. Accelerated sol gelation was also observed for calcium acetate (Yu et al. [12]), as well as lithium citrate (Maçon et al. [30]), due to the basic nature of these salts. A very recent study of Bossard et al. described Ca(OH)_2_ as a suitable precursor for Calcium(II) incorporation in silica-networks at room temperature [13].

In 2016, we presented a hybrid biomaterial consisting of a sol-gel derived glass and 3- or 4‑armed macromers that were processed to remarkably strong macroporous scaffolds via indirect 3D printing [31]. This processing technique was also employed for other Bioactive Glass Hybrids [32]. Via the incorporation of oligo(d,l-lactide) domains in the organic cross-linker, we got hybrids with a controllable degradation rate enduring over 12 months [33]. Unfortunately, these initial glass formulations did not present convincing bioactivity [31]. In the next step, we developed bioactive hybrid glass scaffolds fabricated from ternary silica sol 65S30C containing 30 mol% Calcium(II) from CaCl_2_ and 5 mol% triethyl phosphate (TEP) [34]. Homogeneous Calcium(II) distribution was found to depend on high contents of organic cross-linker (60%) and was attributed to binding to polar ether functionalities in the oligoethylene glycol containing three armed cross linkers. Further investigations showed, however, that these scaffolds became mechanically weak within a couple of days after incubation in aqueous media. 

Hence, the aim of this study was to develop mechanically stable hybrid scaffolds that show a controlled Calcium(II) release to create implant bioactivity and support bone formation. With regard to material cytocompatibility, a burst release of Calcium(II) as well as a pH change should be avoided.

We therefore limited the amount of CaCl_2_ in the scaffolds to a range of 1–10% and investigated Calcium(II) release from the scaffolds. We hypothesised, that small Calcium(II) amounts may be physically entrapped in a hybrid silica matrix which may be favoured via interactions with the polar functionalities of the applied organic cross-linkers [34]. In a second approach, we used a different Calcium(II) source, bioactive 45S5 glass microparticles (Vitryxx^®^), which required a change in the curing protocol. We investigated scaffold morphology and mechanical performance, bioactivity in simulated body fluid (SBF), Calcium(II) release, as well as changes in compression strength over 12 weeks of incubation. Finally, an in vitro cell culture with osteoblast-like SaOS-2 cells and human bone marrow derived mesenchymal stem cells (hMSCs) served to evaluate the biocompatibility of the selected scaffold formulation, including cell attachment, proliferation and osteogenic differentiation.

## 2. Materials and Methods

### 2.1. Materials

Calcium chloride dihydrate and proteinase K were purchased from AppliChem (Darmstadt, Germany). Deuterated dimethyl sulfoxide (d_6_-DMSO) and deuterated tetrahydrofuran (d_8_‑THF) were purchased from Armar Chemicals (Leipzig, Germany). 3‑isocyanatopropyltriethoxysilane (ICPTES) was purchased from Fisher Scientific (Schwerte, Germany). Polycaprolactone (PCL, Mn 45,000), pentaerythritol ethoxylate PETEO 797 (average Mn 797) and Dulbecco’s modified Eagle’s medium (DMEM), McCoy’s medium, dexamethasone, b-glycerolphosphate, ascorbic acid-2-phosphate, ALP-substrate and 4′,6-Diamidin-2-phenylindol (DAPI), TEOS, TEP were purchased from Sigma-Aldrich (Seelze, Germany). Tetrahydrofuran (THF) was purchased from VWR International (Dresden, Germany). PicoGreen^®^ and Alexa Fluor^®^ 568 Phalloidin were purchased from life technologies (Darmstadt, Germany). Calcium AS FS assay was purchased from DiaSys (Flacht, Germany). OsteoImage^TM^ Mineralization Assay was purchased from Lonza Bioscience (Basel, Switzerland). Mouse monoclonal osteocalcin antibody G-5, suitable for the detection of the osteocalcin protein of human origin conjugated with Alexa Fluor™ 488 Phalloidin (catalogue number: sc-365797) was purchased from Santa Cruz Biotechnology (Heidelberg, Germany). Bioactive 45S5 glass microparticles (Vitryxx^®^) were purchased from Schott AG (Mainz, Germany). According to the data provided from the manufacturer particle size was d50: (4.0 ± 1.0) µm and d95: ≤20 µm. 45S5 bioactive glass consists of 45% SiO_2_–24.5% Na_2_O, 24.5% CaO, 6% P_2_O_5_ (wt%).

### 2.2. Methods

#### 2.2.1. Template Fabrication

Hybrid glass scaffolds were produced from PCL templates by means of 3D-printing technique using a Bioscaffolder^®^ (SYSENG) and regenHU 3DDiscovery^TM^, as described by Hendrikx et al. [31]. Briefly, the template material was melted at 71 °C and extruded through a hollow needle (0.178 mm inner diameter) in a meandering pattern to produce a cylinder with a layer grid structure. Two layers of PCL were extruded on top of each other. The printing orientation was then changed by 90°. The printing proceeded in this way until a template of high definition was built. Line spacing in the x-y level was set to 0.43 mm and in z-level 0.14 mm. Printed templates had a closed surface and open pores from both sides. Dimensions of build template cylinder were measured 7.9 × 8 mm (d × h), inner diameter 7 mm. 

#### 2.2.2. Silica Sol Synthesis

Silica sol was produced according to the modified protocol of Hendrikx et al. [31]. Briefly, the required amount of calcium salt CaCl_2_ × 2H_2_O was dissolved in deionized water and 1 mL of 1 M HCl. Immediately after that, a fixed volume of 12.107 mL TEP was added. Subsequently, 64.3 mL of TEOS was added to yield a concentration of 2.85 M and a molar ratio of water and TEOS (R-ratio) of 2.2. After a transparent solution was obtained, the solution was stirred at 55 °C for 18 h. Finally, ethanol was evaporated at 70 °C in a rotary evaporator under reduced pressure for 6.25 h. Depending on the amount of added calcium salt, following sol formulations were prepared: 0C10P, 2.5C10P, 5C10P and 10C10P (Table 1).

#### 2.2.3. Cross-Linker Synthesis

Triethoxysilane-terminated cross-linkers were produced in a bulk reaction according to the method described by Messori [35] and Hendrikx [31]. The silanization agent 3‑isocyanatopropyltriethoxysilane (ICPTES) was mixed in molar ratio of 4:1 with pentaerythritol ethoxylate P797 (average Mn 797). The reaction was carried out in a sealed 20 mL snap-cap vial. Briefly, P797 was weighed on an analytical balance and the respective amount of ICPTES added. Then, both components were mixed with a magnetic stirrer at 900–1000 rpm at 120 °C in a triethylene glycol bath until a clear solution formed. After the homogenization step, the rotation was reduced to 450–500 rpm and kept for 3 h. The reaction temperature was controlled with a contact thermometer coupled hotplate. The reaction product was cooled down to room temperature before it was used for the hybrid synthesis.

#### 2.2.4. Hybrid Scaffold Production

Organic-inorganic hybrid scaffolds were prepared with 30% of organic cross-linker with the help of the SpeedMixer DAC 150.1 FVZ-K (Hauschild GmbH & Co. KG, Hamm, Germany). Silica sol and organic cross-linker were weighed in a mixing polypropylene cup (10 mL, Hauschild GmbH & Co. KG, Hamm, Germany) and subsequently mixed at 2000 rpm 2 cycles for 2 min. Right afterwards, 3D-printed PCL templates (14 pieces) were put into the cup with the hybrid sol and firmly closed for the casting step, carried out at 1000 rpm performed in 2 cycles for 2 min. The hybrid sol was allowed to cure at room temperature in 48-well plates of polystyrene (PS) with a PTFE bottom plate for 6 days before the PCL template was leached from the gels with tetrahydrofuran (THF). Hybrid gel scaffolds were washed four times with THF in a glass beaker and then dried in a programmable oven in an open container. Drying of the gels was carried out in the same beaker by loosening the lid to allow gas evaporation and heating according to a three-stage heating schedule Table 2.

#### 2.2.5. Hybrid Scaffold Production with Incorporation of Bioactive Glass (BG) Microparticles 

As with the hybrid formulations with no incorporated particles, components mixing and template filling was performed using the SpeedMixer DAC 150.1 FVZ-K. BG powder was disaggregated in a porcelain mortar shortly before use. Depending on the intended formulation, the required amount of BG powder and organic cross-linker were weighed in polypropylene mixing cup. BG powder particles were homogenously distributed in a cross-linker volume within one mixing step performed at 3000 rpm for 3 min. In the next step, the required amount of inorganic sol was added and the second mixing cycle was carried out at 1000 rpm for 2 min. Filled templates were later placed on a petri dish for the gelation and curing step, which was carried out according to the three-stage heating schedule, Table 2. In the next step, melted PCL residues were dissolved in THF and washed out in a glass beaker. Scaffolds were dried at +40 °C for 7 d as previously described [34].

#### 2.2.6. Scaffold Characterization

##### Scanning Electron Microscopy (SEM) and Energy Dispersive X-ray Spectroscopy (EDX)

For SEM imaging and EDX analysis hybrid glass scaffolds were mounted on aluminium and sputter-coated with gold or carbon, respectively. SEM-images and EDX spectra were recorded on a CS 44 SEM (Cam Scan, Waterbeach, UK) using the acceleration voltage of 20 or 10 keV, respectively. 

##### Mechanical Testing

Mechanical properties of scaffolds in dry and wet state were tested on a ZWICK Z010 equipped with a 10 kN load cell (Zwick GmbH & Co. KG, Ulm, Germany). Specimens were tested between two parallel plates with a crosshead speed of 1 mm/min and a preload of 5 N until scaffold failure. Compressive strength was calculated from the maximum compressive load and the cross-sectional area of the sample before the tests. For calculation of compressive strength, the diameter of the specimens before compression was used.

##### Characterization of Scaffold Degradation and Calcium(II) Release 

In vitro scaffold degradation was investigated according to the protocol of Kascholke et al. [33]. TRIS-buffer solution (50 mM) was used as the incubation medium. Firstly, the initial dry weight (w_0_) of the scaffold was determined. Subsequently, specimens were immersed in a approx. 5 mL volume of TRIS–buffer (50 mM) under reduced pressure (50 mbar) for approx. 10 min for air displacement. Afterwards, scaffolds were transferred in 10 mL glass vials and filled with 5 mL of TRIS–buffer. Glass vials were tightly sealed with a plastic screw lid. The degradation study was performed at 37 °C on an orbital shaking at 100 rpm. Medium change was performed on day 1 and 3 of incubation and then weekly. pH measurements were performed with the help of a glass electrode Orion 3 Star pH meter (Thermo Fisher Scientific, Schwerte, Germany) equipped with a N6000 BNC electrode (SI Analytics, Weilheim, Germany). The reduction of the mechanical strength of the scaffolds was investigated at selected time points. Morphological changes to materials were monitored with a stereomicroscope Hund Wetzlar stereomicroscope SM33 equipped with a fibre light source FLQ 150 (Helmut Hund GmbH, Wetzlar, Germany). Incubated scaffolds were dried in a vacuum until reaching a constant dry weight (w_dry_). Weight loss was calculated. 

##### Quantification of Released Calcium(II) 

Calcium(II) ion release was measured during scaffold incubation employing a commercial Ca AS‑FS assay (DiaSys Diagnostic Systems GmbH, Holzheim, Germany). The measurement was performed according to the manufacturer protocol. Briefly, 10 µL of sample solution was mixed with 1 mL of reagent solution in a 1.5 mL Eppendorf tube, immediately vortexed and incubated at the room temperature for 5 min. After the incubation step, the coloured solution was again vortexed and transferred into disposable cuvettes to perform the measurement. For the quantification and concentration calculations, a reference solution supplied with the assay-kit was used. Intensity measurements were performed photometrically (Genesys 6, Thermo Fisher Scientific, Madison, WI, USA). 

Additionally, calcium, sodium, phosphorous and silica ion concentrations were determined using an inductively coupled plasma-optical emission spectrophotometer (ICP-OES; SPECTRO Analytical Instruments GmbH, Kleve, Germany). Cell seeded hybrid glass scaffolds were incubated for 3, 6, 8, 10 and 13 d in triplicates in 24‑well plate containing 2 mL cell culture medium. The medium supernatant was removed and exchanged by 2 mL of fresh medium at every measurement point. Aliquots of 0.5 mL were diluted 1:10 in deionized water. Obtained values were calibrated against standards. The presence of the culture medium in the samples was verified to have no effect on the slope of the calibration.

##### Bioactivity Testing in Simulated Body Fluid (SBF)

The bioactivity testing of hybrid glass scaffolds was carried out according to the protocol described by Hendrikx et al. [34]. Briefly, dried hybrid glass scaffolds (dimensions 3 × 7.9 mm—h × d) were divided into two halves and then sterilized at 160 °C for 2 h. In order to wet the complete material surface with testing solution, specimens were incubated in a small amount of SBF at reduced pressure (50 mbar). SBF solution was prepared following the protocol of Kokubo [36]. Each scaffold half was immersed in 45 mL of SBF at 37 °C on an orbital shaker set to 120 rpm [37]. The ratio of scaffold surface area to volume of the incubation solution was 1 cm^2^ per 12 mL. At selected time points (d2, d7 and d14) the incubation medium was discarded and specimens were rinsed with demineralized water and dried in a 48-wellplate before further investigations.

##### Cell Culture

For cell culture tests, scaffolds derived from 0C10P, 0C10P + BG-MP (incorporating 45S5 glass microparticles) and 2.5C10P hybrid sols were produced. Scaffolds were preincubated for 24 h in culture medium (CM SaOS-2: McCoy’s 5a with l-glutamine supplemented with 1% penicillin/streptomycin and 15% foetal calf serum (FCS), CM MSCs: DMEM low glucose). Cell seeding was performed according to the protocol described by Lieb et al. [38]. Briefly, scaffolds were dynamically seeded with 300,000 SaOS-2 cells for 24 h in CM using spinner flasks. Immediately afterwards, seeded scaffolds were transferred in 24-well plates filled with 2 mL of osteogenic medium (OM, DMEM low glucose, supplemented with 1% penicillin/streptomycin, 10% foetal calf serum (FCS), 255 nM dexamethasone, 10 mM β–glycerolphosphate and 252 µM ascorbic acid-2-phosphate) and incubated at 37 °C and 5% CO_2_ on an orbital shaker (1 cm orbit, 117 rpm). Medium change was performed three times a week. Scaffolds without cells served as further controls. Cell number was determined using Quant-iT^™^ PicoGreen^®^ dsDNA Assay Kit (Invitrogen) on days 2, 3 and 7 as described previously by Schroeck et al. [39]. The osteogenic cell differentiation was investigated by alkaline phosphatase (ALP) measurements performed according to the protocol described by Sedaghati et al. [40]. Briefly, the enzymatic conversion of 4-nitrophenyl phosphate to yellow *p*-nitrophenol catalysed by ALP was measured spectrophotometrically via a kinetic measurement (25 cycles, 5 min each) at microplate reader Synergy H1 (BioTek, Bad Friedrichshall, Germany). Cryosections were prepared after 7 and 14 days of culture using a Leica cryostat CM 1950. The 10 µm thick sections were prepared and fixed to a glass slide with a transparent double-sided adhesive film and stained with DAPI/Phalloidin and Osteoimage™ to determine the cell distribution and spreading, mineralization and matrix formation. Osteogenic differentiation was examined by staining for osteocalcin (OCN). For immunofluorescent staining, osteocalcin antibody (G-5) suitable for the detection of the osteocalcin protein of human origin was used. For the visualization, a conjugate with Alexa Fluor™ 488 Phalloidin was used (mouse monoclonal osteocalcin antibody G-5, Santa Cruz Biotechnology, 200 U/mL stock solution diluted 1:100 in blocking solution).

#### 2.2.7. Statistical Analysis

Differences between groups were calculated using one-way ANOVAs and in conjunction with Tukey’s HSD Post Hoc test (GraphPad Software V.6.01, La Jolla, CA, USA). Results which were statistically significantly different were indicated by a single symbol, star or hash key, (*) for *p*-values below 0.05 or by doubled symbols (**) for *p*-values < 0.01. Data were plotted as arithmetic mean with standard deviation as error bar. 

## 3. Results

### 3.1. TEP as Solvent for Hybrid Sols

**Figure A1:**
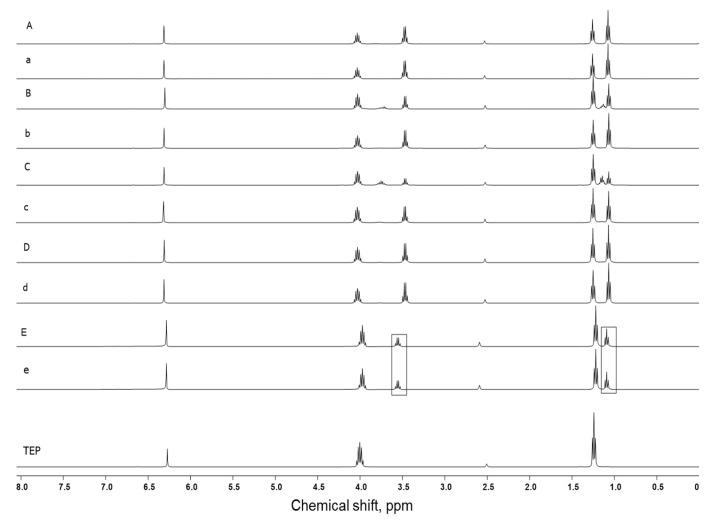
Results of ^1^H-NMR analysis of TEP hydrolysis catalyzed by 1 M HCl (**A**); 0.01 M HCl (**B**), 0.1 M HCl (**C**), 1 M NaOH in presence of EtOH (**D**) and by 19 M NaOH (**E**). Spectrum of TEP served as a control. Data was recorded at the time points: 3 h (small letters) and 30 h (capital letters).

**Figure A2:**
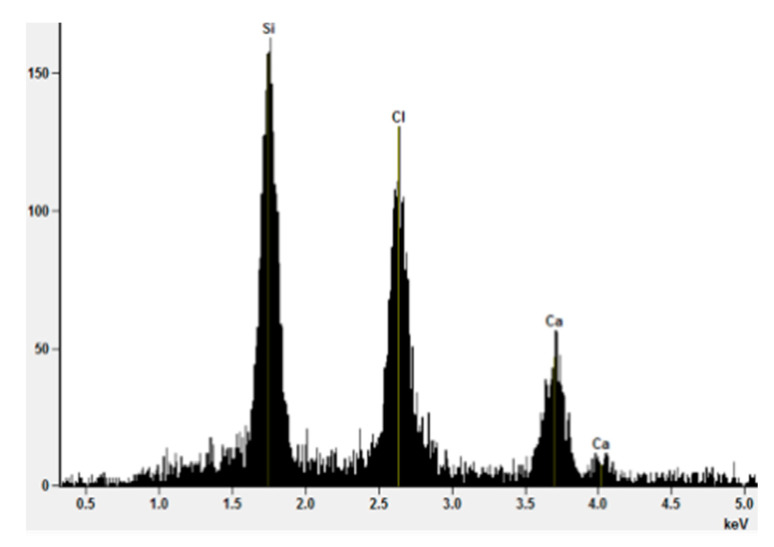
EDX spectra of the surface of hybrid glass scaffolds derived from 65S30C silica sol.

**Figure A3:**
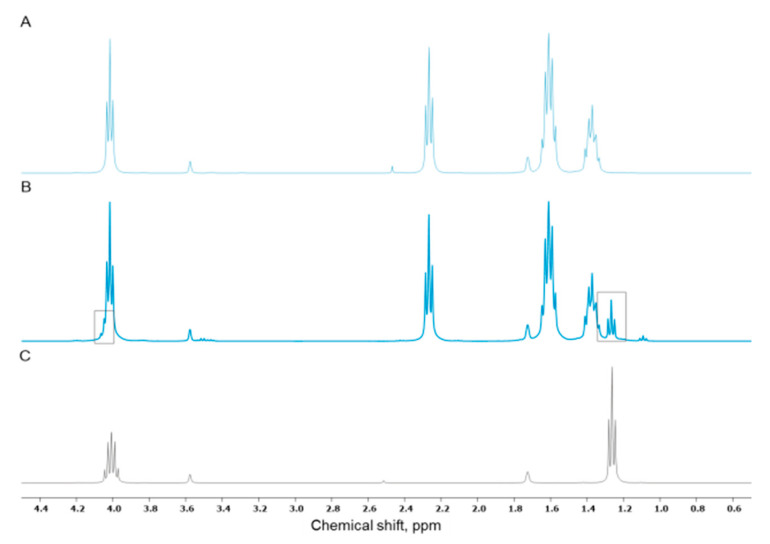
^1^H-NMR spectra. (**A**) PCL. (**B**) THF fraction containing dissolved PCL and TEP after template dissolution. (**C**) TEP.

TEP in bioactive glasses is considered a P_2_O_5_ precursor in silica networks. Several references state a successful incorporation even without thermal treatment [41,42,43,44]. We and others before us [45,46,47,48,49], however, found an extremely low susceptibility to acid-catalysed hydrolysis (Figure A1). Consequently, we did not find any phosphor with EDX on the scaffold surface after template leaching in THF (Figure A2 and Figure A3). Since TEP nonetheless led to improved mechanical performance compared to formulations without TEP, we revised the role of TEP and considered it as a silica sol solvent, that allows to control sol viscosity, supports a homogeneous incorporation of organic cross-linker during the hybrid synthesis and prevents premature sol gelation. Due to the almost complete TEP removal during the template leaching, any cytotoxicity that may be related to this reagent should not compromise the cytocompatibility of the final material. To the best of our knowledge, we present for the first time a silica sol synthesised with 10 mol% TEP (10P) where TEP was used as a silica sol solvent without addition of any other organic solvent.

### 3.2. Incorporation of Calcium(II) from CaCl_2_ and Bioactive Glass Microparticles (Vitryxx^®^) 

**Figure A4:**
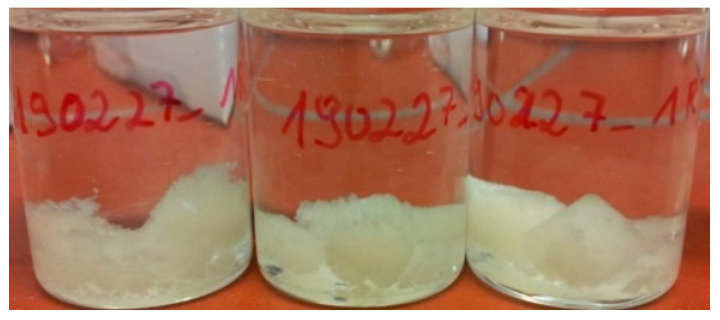
Scaffolds containing BG microparticles cured at room temperature for 6d and collapsed upon the template leaching with THF.

**Figure A5:**
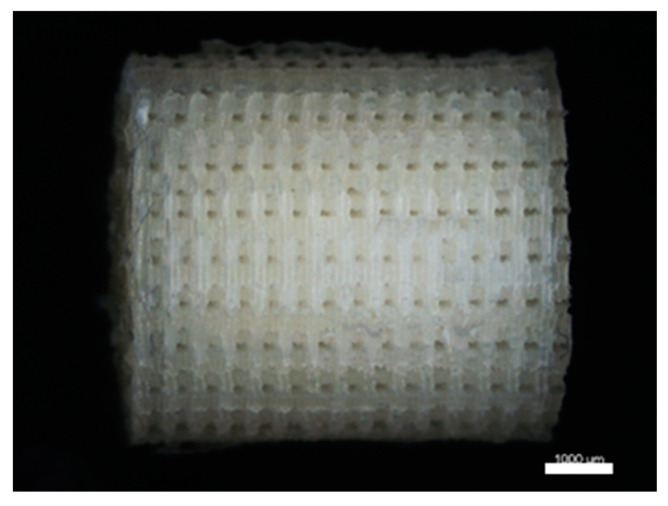
Mechanically stable scaffold containing BG microparticles cured following the three-stage heating protocol for 6d and dried at 40 °C for 7d. Scale bar represents 1000 µm.

TEOS-derived sols containing 30% organic cross-linker P797-Si served as common basis for the fabrication of Calcium(II)-containing hybrid glass scaffolds. Calcium(II) releasing silica sols either contained CaCl_2_ with Calcium(II) concentrations between 1 (1C) and 10% (10C) (1C10P to 10C10P) or bioactive glass microparticles (0C10P + BG‑MP). For the incorporation of CaCl_2_, we followed a protocol involving curing for six days at room temperature; this protocol turned out inefficient for the incorporation of BG-MP. Following the six day curing protocol, scaffolds containing BG-MP collapsed upon the template leaching step (Figure A4). We hypothesized, that BG-MP might disturb homogeneous hybrid matrix formation due to the elevated pH values in the BG-MP vicinity causing missing cross-linker integration and possibly phase separation. Increased pH is known to support the condensation of free silanol groups [50] and accelerate gelation [13]. Consequently, we had to establish a new curing protocol overcoming this inhomogeneity in network formation. We chose to increase the curing temperature to 60 °C in the first step (Table 2) in order to add activation energy to increase condensation velocity and to overcome phase separation [50,51]. In fact, although the temperature exceeded the melting temperature of the PCL template, this did not interfere with the formation of a regular porous structure in the final scaffolds (Figure A5). When we used the same protocol for the BG-MP-free formulations, template breakdown was faster than gelation of the hybrid sol. Thus, when BG-MP free scaffolds were processed, this 3-step thermal treatment protocol was only applied for the drying step after gelation and template removal. 

### 3.3. Scaffold Strength

**Figure 1:**
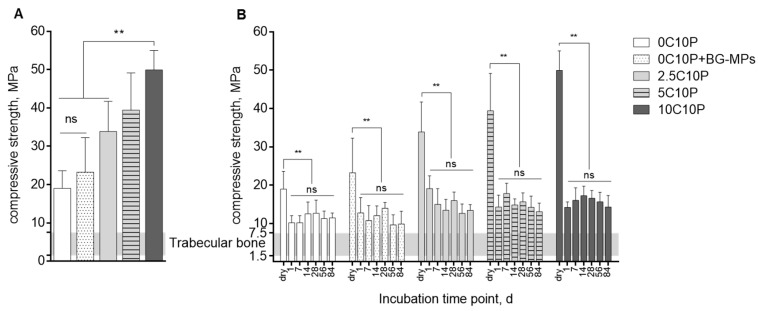
(**A**) Compressive strength values of hybrid glass scaffold measured in dry state (*n* = 6) after scaffold fabrication. (**B**) Compressive strength values of hybrid glass scaffolds as a function of immersion time in TRIS, tested under wet conditions (*n* = 6): 0C10P, 0C10P+BG‑MPs, 2.5C10P, 5C10P, 10C10P. Gray area marks compressive strength range of human trabecular bone [52]. Compressive strength values measured in dry state are integrated in order to show the extent of changes in compressive strength after incubation in TRIS buffer. Formulation names: 0 to 10 C: 0 to 10% Calcium(II) by addition of CaCl_2_, + BG MPs: addition of Vitryxx bioactive glass microparticles, 10P: addition of 10% triethyl phosphate (TEP). (**) for *p*-values < 0.01, (ns) for not significant.

Scaffold mechanical stability and scaffold behaviour under compression were evaluated by uniaxial compression tests. Figure 1A shows compression strength of scaffolds dried following a 3-step drying protocol (Table 2). 

Overall, fabricated hybrid glass scaffold formulations showed high mechanical stability in dry state with the compressive strength values clearly higher than the compressive strength range reported as 1.5–7.5 MPa for trabecular bone [53,54]. The highest compressive strength values up to 56 MPa were measured for scaffolds produced from 10C10P hybrid sol, whereas the lowest values were found for 0C10P without incorporated Calcium(II). Incorporation of BG-MP had no significant effect on scaffold mechanical stability. Mechanical strength of all tested scaffold formulations, however, showed quite high standard deviations. This might be explained by filling defects due to gas adsorption on hydrophobic PCL templates. Additionally, structure flaws may also originate from ethanol evaporation during hydrolysis and condensation reactions as well as gel syneresis accelerated by elevated temperatures applied during the drying step [17,55].

### 3.4. Scaffold Degradation upon Incubation in Aqueous Medium 

**Figure A6:**
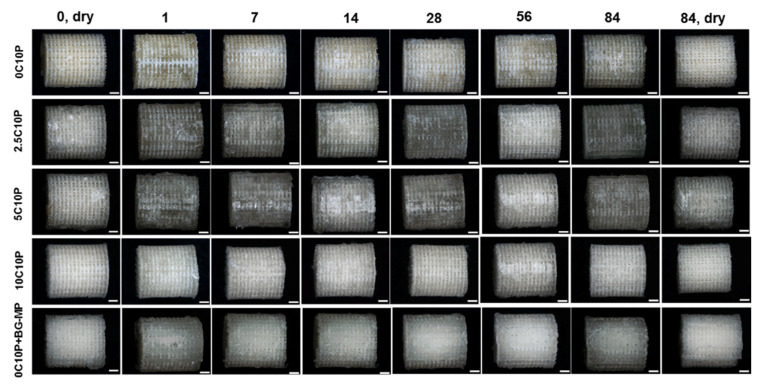
Stereomicroscopic images of wet and dried scaffolds at selected incubation time points in TRIS-solution (50 mM). Scale bar represents 1000 µm.

Changes in mechanical properties caused by immersion in TRIS-buffer for a period of 84 days are illustrated in Figure 1B. One day after immersion, we observed a significant decrease in scaffold compression strength compared to the values measured for the dry state for all formulations. This can be partially referred to the plasticizing effect of water [56,57,58]. Additionally, we registered a weight loss that correlated with the scaffold composition (Table A1) and indicates that dissolution of precipitated CaCl_2_ contributed to the remarkable initial decrease of the compressive strength especially for 5C10P and 10C10P derived scaffolds with high CaCl_2_ content (Table 1) [14,16,29]. This is exemplarily shown for 10C10P, where the drop in compressive strength was found to be 72% whereas that for 0C10P was only 47%. This implies that CaCl_2_ did not incorporate into the silica network [14,59] but recrystallized on the scaffold surface and filled out structural flaws increasing scaffold strength in dry state. No significant changes in compression strength were determined after day 1 suggesting only minor bulk degradation [60] over the incubation time scale of 12 weeks. Apart from the initial drop in scaffold stability, all measured values remained between 10 and 20 MPa which is still beyond that reported for the human trabecular bone [61]. In line with data on mechanical properties, tested hybrid glass scaffold formulations showed no significant changes of scaffold structure during the entire incubation period (12 weeks). Scaffold dimensions (height and diameter) were not significantly changed, suggesting only minor scaffold degradation and swelling (Figure A6).

### 3.5. Bioactivity Testing 

Material bioactivity is described in terms of carbonated hydroxyapatite (HCA) [50] layer formation on its surface; this is important for implants to bond to bone [62] without a distinct boundary [63]. The apatite formation on the surface of a material in simulated body fluid (SBF) is considered as a predictor for bone bonding ability of the material in vivo. A material’s bioactivity is expected to increase with the reduction of time needed for the apatite layer formation on its surface [15]. Surface mineralization starts with a rapid ion exchange between hybrid material and incubation medium [4,54,64], followed by the formation of a silica rich layer with abundant free silanols that act as nucleation sites [11,54,65,66] for calcium phosphate crystals that may eventually coalesce into a coherent layer [67]. 

**Figure 2:**
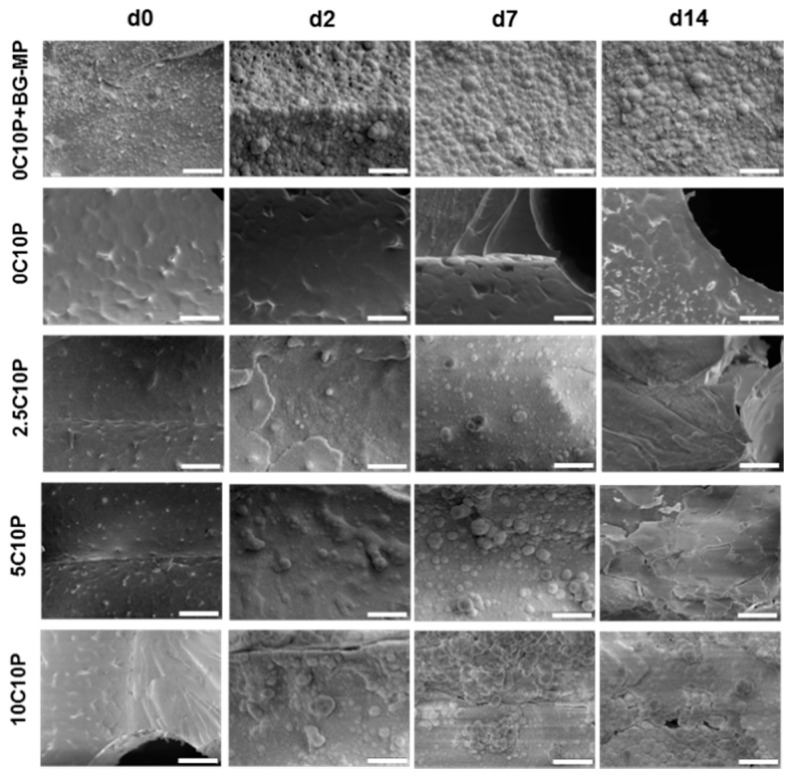
Scanning electron microscopy (SEM)-micrographs of scaffolds from different formulations after 0, 2, 7 and 14 days of immersion in simulated body fluid (SBF). Scale bars represent 25 µm. Formulation names: 0 to 10 C: 0 to 10% Calcium(II) by addition of CaCl_2_, + BG-MPs: addition of Vitryxx bioactive glass microparticles, 10P: addition of 10% TEP.

Scaffold bioactivity was studied by immersion of selected scaffold formulations in SBF and subsequent SEM analysis (Figure 2).

Before immersion, all scaffold formulations showed a homogenous surface with no visible signs of phase separation. Only in SEM images of the 0C10P + BG-MPs formulation could MPs be identified in the hybrid glass surface as homogeneously distributed over the complete scaffold material with no visible aggregates (Figure 2, 0C10P + BG-MP, d0). 

Only minor changes within the first week of incubation were seen for CaCl_2_ containing 0C10P and 2.5C10P scaffolds, whereas the introduction of 5 and 10 mol% soluble Calcium(II) resulted in an oversaturation of SBF solution and mineral layer precipitation on the material surface already after 2 days of incubation [4]. We observed a formation of round shaped mineral with cauliflower morphology and partially needle-like structures, that can be attributed to the apatite-like mineral [15,68,69,70]. Generally, material bioactivity is related to its elemental composition (Si/Ca/P–ratio), textural properties, such as surface morphology, pore structure, pore size and pore volume [3,68,71,72,73,74]. The bioactivity of 0C10P scaffolds observed at day 14 may be therefore attributed to the changed surface textural properties and increased porosity, caused by the leaching of TEP during the template dissolution. 

Similar to the 10C10P formulation, we observed rapid mineral formation on the surface of 0C10P + BG-MP. Since bioactive glass 45S5 is known to show strong bioactivity by alkalization of the surrounding medium [54] and release of Calcium ions, we further investigated both parameters for the BG-MP and after integration into the 0C10P + BG‑MP formulation in TRIS buffer. Furthermore, the presence of P_2_O_5_ groups in the microparticle structure may have supported the scaffold bioactivity [69,75].

### 3.6. Calcium Ion Release and Media pH

Calcium ions are involved in the bone metabolism and are known to play a substantial role during the processes of angiogenesis, growth and mineralization of bone tissue [76]. Continuous Calcium(II) release in the postulated physiologically relevant range of 60–90 mg/L has been shown to support osteogenic cell differentiation [21,22,77,78,79], whereas higher Calcium(II) concentrations of 90–110 mg/L may be toxic for human osteoblasts [19]. We therefore intended to set up hybrid glass formulations that are able to continuously release Calcium(II) in a physiological suitable range to support bone formation. 

**Figure 3:**
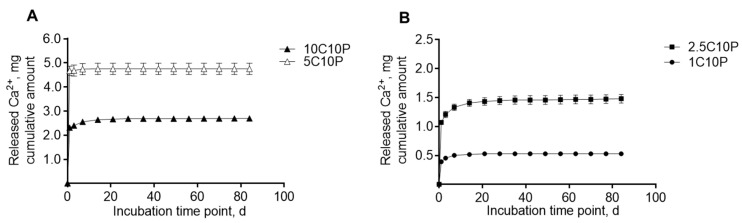
Cumulative Calcium(II) amount released in 5 mL TRIS buffer renewed with each indicated time point over the investigated incubation period of 84 days. (**A**) Release profiles from 10C10P and 5C10P scaffolds. (**B**). Release profiles from 2.5C10P and 1C10P scaffolds. (*n* = 6). Formulation names: 0 to 10 C: 0 to 10% Calcium(II) from addition of CaCl_2_, 10P: addition of 10% TEP.

Calcium(II) release was investigated for all hybrid glass formulations in TRIS buffer in order to prevent Calcium(II) precipitation in the release medium (Figure 3). For all CaCl_2_ containing scaffold formulations (1–10% Calcium(II)), release profiles were dominated by a strong burst release. In case of 2.5C10P, 5C10P and 10C10P (Figure 3), high Calcium(II) concentrations above 90 mg/L that have been described as potentially cytotoxic were reached within the first day of incubation in TRIS buffer, whereas lower concentrations were found for 1C10P (Figure 3B). With decreasing Calcium(II) content in the scaffold formulation, burst release could be reduced but also with only 1% Calcium(II), we still found more than 70% release within one day (Table 3). This means, that formulations with only 30% organic cross‑linker P797 were not suitable to sufficiently bind Calcium(II) after incubation in aqueous medium. 

**Figure 4:**
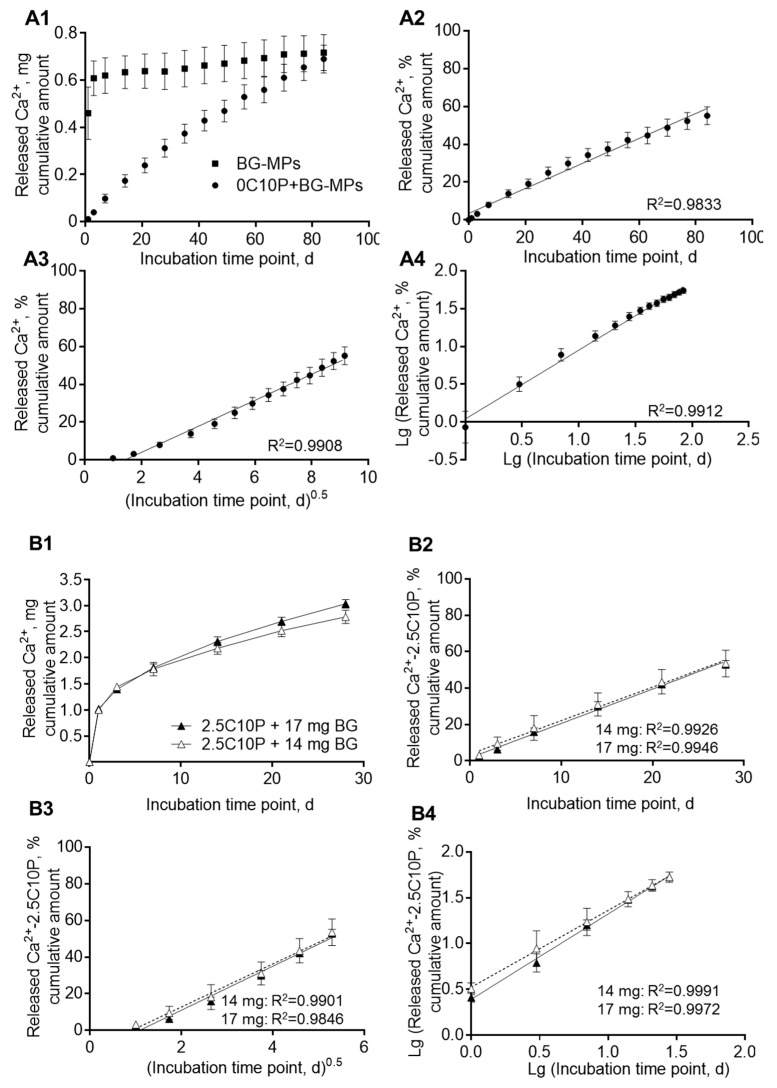
Cumulative Calcium(II) amount released in 5 mL TRIS buffer renewed with each indicated time point over the investigated incubation period of 84 (**A**) or 28 days (**B**). (**A1**) 0C10P-derived scaffolds with incorporated 7 mg bioactive 45S5 glass microparticles (BG-MPs) in comparison to the correlative amount of 45S5 bioactive glass microparticles. (**A2**) linear fit; (**A3**) linear fit to Higuchi model, (**A4**) linear fit to Korsmeyer-Peppas model. 100% Calcium(II) were assumed to be equal to the maximal Calcium(II) contained in 7 mg BG-MP. (**B1**) 2.5C10P-derived scaffolds with incorporated 14 and 17 mg BG-MPs. (**B2**), linear fit, (**B3**) linear fit to Higuchi model, (**B4**) linear fit to Korsmeyer-Peppas model. In (**B**) (**B2**–**B4**), 100% Calcium(II) were assumed to be equal to the maximal Calcium(II) contained in 14 or 17 mg BG-MP. In (**B**) for (**B2**–**B4**), the cumulative Calcium(II) amount released from 2.5C10P-derived scaffolds was subtracted in order to show the controlled release originating from the added BG MPs. Formulation names: 0 to 10 C: 0 to 10% Calcium(II) from addition of CaCl_2_, + BG MPs: addition of Vitryxx bioactive glass microparticles, 10P: addition of 10% TEP.

In contrast, scaffolds incorporating BG-MP showed a prolonged Calcium(II) release (Figure 4). For formulation 0C10P + 7 mg BG-MP, we observed an almost linear release over 12 weeks, with a release rate of approximately 12 µg of Calcium(II)/day (Figure 4A, A and B in the Table 4). When we incubated the same amount of naked BG-MP, we found burst release with 65% Calcium(II) release after 1 day and 85% after 3 days of incubation in TRIS buffer (Figure 4A, Table 4). This indicates that Calcium(II) release by ion exchange from BG particles embedded in the hybrid glass matrix is effectively sustained. This can be caused by reduced equilibration of Calcium(II) concentration and pH at the accessible surface of the embedded particles with the surrounding buffer [80]. For the incorporation of higher amounts of microparticles–14 and 17 mg, the 2.5C10P sol formulation was used. This was necessary in order to prevent fast gelation that would have interfered with the processing of the sol into scaffolds. As expected we observed a burst release of Calcium(II) due to the incorporated 2.5% Calcium(II) from CaCl_2_, followed by a prolonged release (Figure 4B). In order to separate the release generated by CaCl_2_ from that caused by the integration of BG-MP, we subtracted the release determined for the 2.5C10P formulation (Figure 3B, Table 3) from the BG-MP containing formulations. Within 4 weeks of incubation, we observed for the formulations with higher BG-MP content a decreasing Calcium(II) release rate. Nonetheless, even in the fourth week of incubation, we measured a release rate of 38 and 48 µg/d (A in the Table 4). This means a 4 to 5fold higher release of Calcium(II) from the formulations containing twice (14 mg) or 2.5 fold (17 mg) the amount of BG-MP compared to the formulation with 7 mg. In order to investigate the release mechanisms, we calculated different release profiles according to Higuchi for matrix-controlled diffusion (Figure 4A3,B3) and the Korsmeyer-Peppas model (Figure 4A4,B4). All models, however, showed strong correlations with correlation coefficients of more than 98%. 

**Figure 5:**
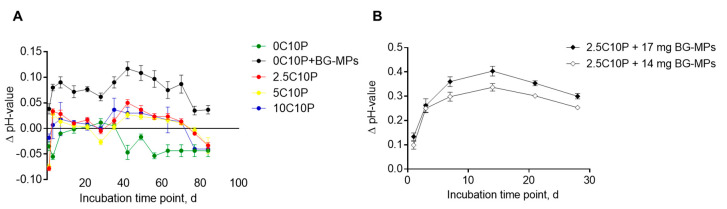
pH-changes of incubation medium registered during scaffold incubation. Values are represented as Δ pH values in relation to the pH-value of TRIS buffer solution (50 mM) (7.39 ± 0.03) measured at selected incubation time points. (**A**) 0C10P+BG‑MPs (black), 2.5C10P (red), 10C10P (blue), 5C10P (yellow), 0C10P (green). (**B**) 2.5C10P+BG‑MPs (14 mg), 2.5C10P+BG‑MPs (17 mg) (*n* = 6). Formulation names: 0 to 10 C: 0 to 10% Calcium(II) from addition of CaCl2, + BG MPs: addition of 45S5 bioactive glass microparticles, 10P: addition of 10% TEP.

Observed ion release from incubated scaffolds correlated with the pH-changes of TRIS buffer incubation solution (Figure 5).

Scaffolds without incorporated BG-MPs led to a slight pH shift of TRIS buffer in a physiological pH‑range from 7.3 to 7.5. After more than 30 days, we observed a slight shift to the acid range. Significantly higher pH value changes were measured for hybrid glass scaffolds containing BG-MPs, with the solution alkalization correlating to the amount of incorporated BG-MPs. For the 0C10P+BG-MPs, containing 7 mg of BG-MP, TRIS buffer pH never exceeded 7.53 during the incubation time (Figure 5A). In contrast, for formulations with twice the amount of BG-MP (2.5C10P+BG-MP), we found an increase in pH to more than 7.7 (Figure 5B). Due to this clear increase in pH, we considered the formulations with 14 and 17 mg of BG-MPs as less suitable for cell culture experiments although the released amount of calcium was more promising and continued with the 0C10P+BG-MP formulation. 

### 3.7. Cell Culture Experiments

**Figure 6:**
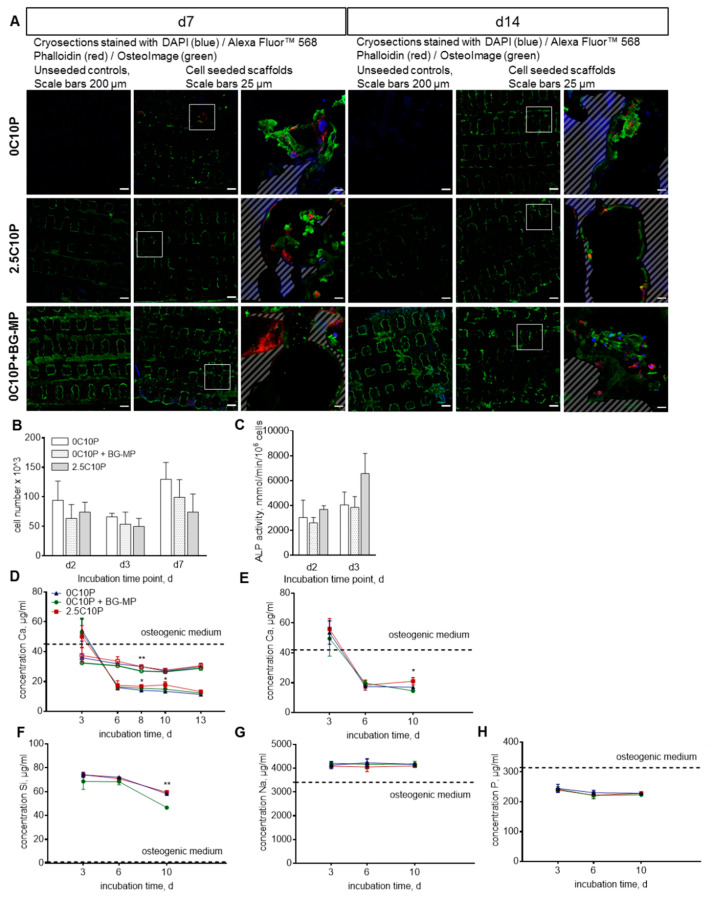
(**A**) Microscopic images of cryosections of hybrid glass scaffolds, formulations 0C10P, 0C10P-BG-MPs and 2.5C10P, seeded with SaOS-2 cells, cultured for 7 and 14 days and stained with DAPI (blue)/Alexa Fluor™ 568 Phalloidin (red)/OsteoImage (green) staining. Unseeded scaffolds served as a control. White squares mark the approximate location of magnified images. Hatched areas mark scaffold material in the cross sections. (**B**) Development of cell number over 7 days and (**C**) ALP activity on day 2 and 3 with *n* = 4. d2, d3 and d7 represent time passed after scaffold transfer to osteogenic medium. 0C10P, 0C10P+BG‑MPs, 2.5C10P. (**D**) Calcium(II)-concentration measured in cell culture medium upon scaffolds incubation. Closed symbolize samples seeded with cells. Open symbols represent unseeded samples and served as control with 0C10P, 0C10P+BG‑MPSs and 2.5C10P. Significantly lower Calcium(II) concentration in medium was found on day 8 for 0C10P-BG-MPs scaffolds in comparison to 0C10P and 2.5C10P scaffolds; and on day 10 for 0C10P-BG-MPs scaffolds in comparison to 2.5C10P scaffolds. Calcium (**E**), silicon (**F**), sodium (**G**) and phosphorous (**H**) ion concentrations released from hybrid glass scaffolds in cell culture medium determined by ICP-OES. Significantly lower Calcium(II) concentration in medium was found on day 10 for 0C10P-BG-MPs scaffolds in comparison to 2.5C10P scaffolds; and significantly lower silicon ion concentrations found on day 10 for 0C10P-BG-MPs scaffolds in comparison to 0C10P and 2.5C10P scaffolds. Medium change was performed every 2 or 3 days (*n* = 3).

**Figure 7:**
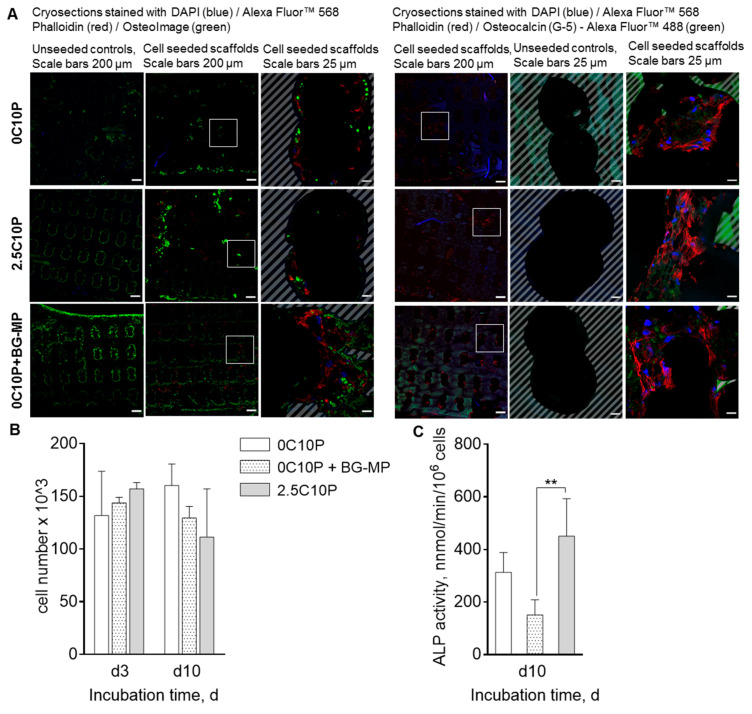
(**A**) Microscopic images of cryosections of hybrid glass scaffolds, formulations 0C10P, 0C10P‑BG-MPs and 2.5C10P, seeded with hMSCs, cultured for 21 days and stained with DAPI (blue)/Alexa Fluor™ 568 Phalloidin (red)/OsteoImage (green) or DAPI (blue)/Alexa Fluor™ 568 Phalloidin (red)/Osteocalcin (G-5)-Alexa Fluor™ 488 (green). Unseeded scaffolds served as control. White squares mark the approximate location of magnified images. Hatched areas mark hybrid glass scaffold. (**B**) Development of cell number over 10 days and (**C**) ALP activity at day 10 with *n* = 4. d0 represents scaffold transfer to osteogenic medium after scaffold seeding. d3 and d10 represent time passed after scaffold transfer to osteogenic medium. 0C10P, 0C10P+BG‑MPs, 2.5C10P. Significant differences for ALP-activity on day10 are shown in comparison to the scaffold formulation containing BG-MPs. (**) for *p*-values < 0.01.

Selected scaffold formulations 0C10P, 0C10P+BG-MPs and 2.5C10P were dynamically seeded with 3 × 10^5^ SaOS-2 cells (Figure 6) and MSCs (Figure 7). Cells attached to the material and infiltrated the pore network of seeded scaffold. For SaOS‑2 cell culture, cell number remained unchanged from d2 to d3; thereafter, cells proliferated (Figure 6B). No significant differences were detected between the different scaffold materials, indicating good cytocompatibility independent of the scaffold formulation. Cells seeded on 2.5C10P scaffolds showed a trend for higher ALP activity at d3 in comparison to both the other formulations (Figure 6C). On day 7, we found mineralized extracellular matrix around the cells for all selected hybrid glass scaffold formulations. Mineralization further increased during the following 7 days. For the control scaffolds that have been incubated in cell culture medium without cells, most prominent mineralization with an even distribution throughout the inner pores of the scaffold was found in 0C10P+BG-MP, whereas less mineral formation was found on 2.5C10P and almost no mineral staining was found for 0C10P scaffolds during the first 2 weeks (Figure 6A). 

Since Calcium(II) concentration is crucial for osteogenic differentiation, we determined the Calcium(II) level in the supernatant of control scaffolds over a period of 2 weeks. Until day 10, Calcium(II) concentration decreased far below the concentration provided by the medium composition (Figure 6D), indicating mineral deposition on the scaffold surfaces at the expense of free Calcium ions. Lowest Calcium(II) concentrations were found in presence of 0C10P+BG-MP, whereas 2.5C10P showed significantly higher values on day 8 and day 10. A clearly different development of Calcium(II) concentration was found when the scaffolds were seeded with osteoblast-like SaOS-2 cells. On day 6, when these cells started to mineralize their extracellular matrix [81], we found a strong drop in Calcium(II) concentration. During the following days, slightly lower Calcium(II) concentrations were found for 0C10P+BG-MP than for 2.5C10P which were confirmed by ICP-OES data for day 10. In addition, we investigated the concentration of silicon, sodium and phosphorous ions in the medium of SaOS-2 cells seeded scaffolds. Besides the lower Calcium(II) concentration, ICP-OES data showed a reduced silicon ion concentration on day 10 for cell-seeded hybrid glass scaffolds containing BG-MPs whereas no difference in sodium or phosphorous was found. 

In order to investigate the material effect with more relevant cells, we seeded hMSCs on the different scaffolds and observed proliferation and differentiation. Cell number remained unchanged from d3 to d10 (Figure 7B). On day 10, ALP activity was significantly lower on 0C10P+BG-MP than on 2.5C10P scaffolds (Figure 7C). After 21 days, we analysed mineral deposition and cell distribution on scaffolds via histological analysis. Cells were homogeneously distributed over the cross sections of the different scaffold types. Seeded cells formed a mineralized extracellular matrix, observed at day 21 of cell culture. Osteogenic differentiation of MSCs was additionally examined by staining for Osteocalcin (OCN) (Figure 7A). Cells cultured on all hybrid glass formulations stained positive for OCN, an osteoblast specific marker. No obvious differences were found concerning the histological characterization of the cells.

In case of 0C10P- and 2.5C10P blank scaffolds (Figure 7), we observed less mineral formation on 0C10P than on 2.5C10P scaffolds on day 21. Scaffolds containing BG-MP, however, carried a thick layer of hydroxyapatite-like mineral evenly distributed throughout the scaffold inner pores (Figure 7A). Whether this is of relevance or not needs to be determined by in vivo studies. 

## 4. Discussion

The motivation for this study was to fabricate hybrid glass scaffolds with sustained Calcium(II) release to create bioactive materials. Implants aimed to be used for bone tissue regeneration should possess sufficient mechanical strength comparable to that of the surrounding bone [82]. All tested scaffold formulations in this study showed reasonable compressive strength after fabrication and were able to keep a sufficient stability after the incubation in aqueous medium for a period of at least 12 weeks. Measured compressive strength was in the range of trabecular bone [53,54]. Observed differences in scaffold compressive strength registered in the dry state were attributed to the positive effect of precipitated CaCl_2_, that was not able to enter the silica network, as no calcination step was applied to this hybrid material [14,16,17] but filled out scaffold structural defects. These defect filling CaCl_2_ depositions were obviously easily accessible for the incubation medium and rapidly dissolved upon incubation. This interpretation is supported by the observed strong reduction in mechanical strength of 10C10P and 5C10P formulations after only 1 day of incubation in TRIS buffer. As we observed no significant differences in mechanical strength of scaffolds of the different formulations after day one, we assume that the CaCl_2_ deposition did not change the structure of the hybrid glass scaffolds. 

In contrast to the burst release observed for CaCl_2_ containing formulations, scaffolds with incorporated BG-MPs showed almost linear Calcium(II) release over 84 days. As already reported by others, a continuous Calcium(II) release in a physiologically relevant range of 60–90 ppm [80] may support cell differentiation [21,22,77,78]. Higher Calcium(II) concentrations of 90–110 ppm were shown to be toxic for human osteoblasts [19]. The application of 45S5 bioactive glass micro- and nanoparticles for the delivery of Calcium(II) have been already described in different matrices, including alginate-based films [83] and hydrogels [84,85], as well as composites of polylactic acid (PLLA) and bioactive glass powders [86]. However, to the best of our knowledge, 45S5 bioactive glass microparticles have not been used before for the incorporation into sol-gel derived hybrid glass scaffolds. In order to determine the mechanism for the controlled Calcium(II) release, we applied different models. We fitted the logarithmic cumulative release versus lg(t) and determined the slope n of the linear function which depends on the release mechanism and the geometry of the carrier system. The analysis revealed a value of 0.9 for the whole range of the incubation period, which implies the transport process is anomalous non-Fickian [87,88]. This is in agreement with the assumed Calcium(II) release mechanism from BG-MP via ion exchange processes [80]. Since naked BG-MPs show burst release with most Calcium(II) released within one day, the controlled release from the scaffolds carrying BG-MPs is limited by slow medium exchange around the embedded BG-MP due to limited buffer diffusion through the scaffold matrix. Within the scaffold material, diffusion distances in the hybrid glass matrix are predetermined by the distances between the PCL strands of the lost mould. Resulting matrix strand diameters are about 250 µm, meaning that distances of maximal 125 µm need to be overcome by diffusion. Mesoporous structure of the hybrid glass as well as cracks generated during the drying process may have accelerated diffusion into and from the matrix. Once Calcium(II) ions left the scaffold strands, the regular pore network with pore diameters of 200 µm in combination with dynamic incubation on a shaker should be suitable for fast transport into the bulk medium. Moreover, the formation of small pores due to degradation, as seen by weight loss upon 84 days of incubation (Table A1), may contribute to the controlled release of Calcium(II) ions. Formulations with higher contents of BG-MP (14 and 17 mg) showed a bimodal Calcium release. Within the first days, we found an initial burst release originating from 2.5% CaCl_2_ and subsequently a continuous release from the BG-MP. More than 50% Calcium(II) originating from incorporated BG-MP were released after 28 days whereas we found only 25% release from the formulation with 7 mg BG-MP after that time. This may indicate that the structure of the scaffolds with higher BG-MP contents has been impaired and allowed for faster diffusion of water to the incorporated BG-MP. In agreement with that, we found significantly lower compressive strength for these formulations compared to 0C10P+BG-MP two week after incubation (data not shown). 

Observed ion release correlated with pH-changes of the incubation solution. With the exception of scaffolds containing BG-MPs, we observed an initial slight acidification of the incubation solution caused by release of acid residues used for sol preparation [33], with a second pH drop after 8 weeks of incubation, probably caused by release of Si(OH)_4_ due to the degradation of (Si‑O‑Si) network [68,89]. In contrast, incubation of scaffolds containing BG-MPs resulted in a fast and notable increase of solution pH due to the release of Calcium(II) and Sodium(I) ions [19,90]. A slight alkalinisation of release media and sustained Calcium(II) release are beneficial with respect to enhanced HCA layer nucleation on the materials surface [4,91]. It is generally accepted, that HCA layer formation supports chemical bonding in vivo [4,91]. 

**Figure A7:**
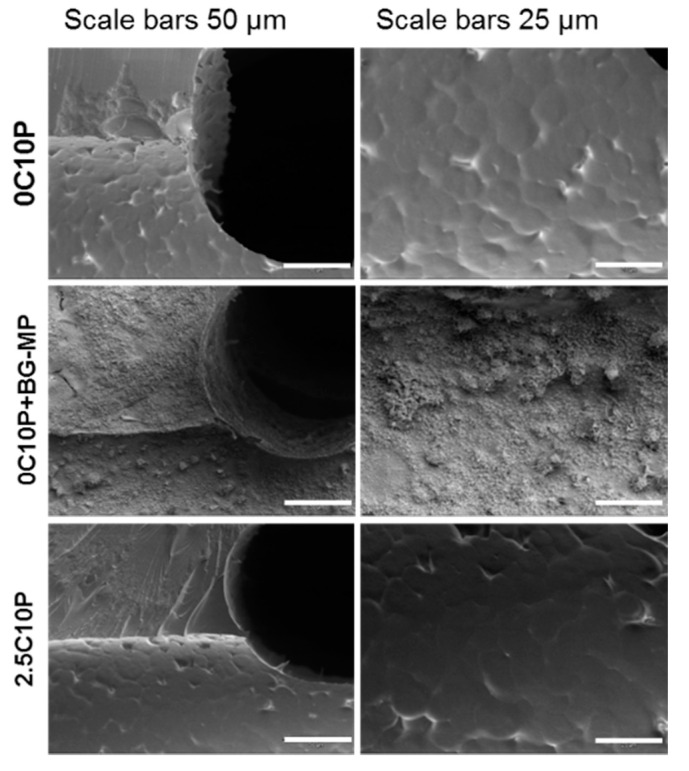
SEM images of scaffold surfaces preincubated in cell culture medium for 24 h.

For in vitro cell culture experiments, we chose 0C10P, 0C10P+BG‑MPS and 2.5C10P scaffolds, due to good stability and Calcium(II) release in a non-toxic range after 24 h preincubation in cell culture medium. Scaffold pre-incubation in serum containing cell culture medium for 24 h prevented initial pH changes [19], provided protein adsorption able to support cell attachment and hydroxyl-carbonate-apatite deposition on the sample surfaces (Figure A7) [89,92,93,94]. SaOS-2 cells and hMSCs seeded on material surface were able to adhere, to proliferate and to deposit apatite-like mineral on the scaffold surface. Incorporation of bioactive glass microparticles led to remarkably increased mineral deposition shown with OsteoImage^®^ staining compared to 0C10P control scaffolds. No significant difference in cell proliferation was observed for the different scaffold materials for SaOS-2 cells and hMSC. Considering osteogenic differentiation, ALP activity, a well-accepted early marker for osteogenic differentiation [95], showed a slight but non-significant increase on 2.5C10P scaffolds in comparison to the BG-containing formulations when SaOS-2 cells were analysed on day 3. Similarly, we observed a significantly decreased ALP activity for hMSC on 0C10P+BG-MP scaffolds compared to 2.5C10P hybrid glass scaffolds on day 10. This may be related to lower Calcium(II) contents that were found in the cell culture medium for the BG formulation. This result was unexpected, as we intended to release additional Calcium(II) to the cell culture medium with this formulation. Although we found a continuous release of Calcium(II) in TRIS buffer, this turned out not to be true for cell culture medium. In cell culture medium, we instead found a thick layer of mineral deposited on the scaffold surface, that obviously consumed the additionally released Calcium(II) [96] and/or prevented its release. Moreover, we found the Calcium(II) content in cell culture medium to strongly decrease when SaOS-2 cells started to mineralize their extracellular matrix on day 6 [21,22,76]. As Calcium(II) is known to support osteogenic differentiation, a reduced amount may have slowed down osteogenic differentiation. In this regard, a preincubation step of two weeks in cell culture medium might help to improve differentiation [97] as the increase in Calcium(II) contents in the medium of non-seeded scaffolds indicates that the formation of the mineral layer has stopped at this time. Additionally, silicon content was found to be decreased in 0C10P+BG-MP on day 10. Silicon is known to support osteogenic differentiation and reduced amount may have inhibitory effects on osteogenic differentiation [20,98]. Further investigations are necessary to test if a combined concept of CaCl_2_ addition and BG-MP may allow for an osteoinductive release of Calcium(II) and silicon.

Literature research offers partially ambiguous data on the effects of 45S5 bioactive glass on osteogenic differentiation of MSCs. As described by Yang et al. [99], the inclusion of 5% 45S5S bioactive glass into PDLLA foams had a positive effect on the differentiation of hMSCs seeded on the materials surface. A good cell differentiation, seeded on 45S5 Bioglass^®^ based scaffolds was shown by Detsch et al. [97]. Contradicting data was published by Reilly et al. [100], who showed no effect of bioactive glass discs on ALP activity of hMSCs in any medium. In the same publication, inconsistent data with high donor variability was shown for the effect of 45S5S bioactive glass dissolution products. As already noted by others [99], the ion exchange occurring in contact with the scaffold may lead to elevated pH-values in the scaffold microenvironment which has been reported to negatively influence osteogenic differentiation. In the study of Monfoulet [101], the ECM mineralization of MSCs was proven to be strongly sensitive to the pH and was fully inhibited at pH ≥ 7.54. However, this finding does not explain the reduced ALP activity of MSCs in our study, since the incorporation of 7 mg BG-MPs in hybrid glass scaffolds led only to a very moderate pH increase (medium pH always below 7.52), measured in TRIS-buffer and no visible difference in colour of pH-indicator containing cell culture medium was observed for the different formulations. This is in agreement with other publications, showing that changes in pH depend on the incubation medium, with TRIS buffer showing stronger changes than DMEM (Thavornyutikarn et al. [90]). The missing increase in Calcium(II) content may be due to immediate precipitation of released Calcium(II) as a mineral layer on the scaffold surface, which was most prominent for the formulation 0C10P+BG-MP [90]. This layer may also have reduced ion release from the scaffold material. 

In this study, we did not preincubate the 45S5 MP containing scaffolds as reported by other studies in order to prevent cytotoxic effect caused by transient pH increases reported for pure 45S5 scaffolds [19,90,97]. This was not necessary, as the pH in DMEM did not increase for the investigated formulations. Nonetheless, the release of Calcium(II) from 0C10P+BG-MP with 7 mg of BG-MP was on a per day basis quite low. Higher release rates, for example, from formulations with a combination of CaCl_2_ and BG-MP, such as the 2.5C10P+BG formulations, may be advantageous and need to be investigated in future studies. 

It remains difficult to predict in vivo effects of BG-MP containing materials, as we did not find any improved osteogenic markers in this study for 0C10P+BG-MP with 7 mg of BG-MP. Nevertheless, the mineral layer formation on the scaffolds as the established measure of bioactivity was convincing. Similarly, other studies on 45S5 bioactive glass did not find positive effects in vitro but in vivo [102] suggesting that bioactive glass supports the bone formation by a more complex mechanism than only the stimulation of MSCs differentiation [100]. 

## 5. Conclusions

In this study, we aimed to prepare mechanically stable sol-gel hybrid glass scaffolds with improved bioactivity. The use of water soluble CaCl_2_ in the scaffold forming hybrid sol resulted in a burst release of Calcium (II) upon incubation in aqueous media. Incorporation of CaCl_2_ in low concentrations in hybrid glass scaffolds may support initial Calcium(II) supply of attaching and differentiating bone forming cells. Calcium(II) contents of 5% and 10% were shown to cause apatite-like mineral deposition on the scaffold surface by super saturation in SBF. Integration of bioactive glass microparticles in the scaffold matrix allows for controlled Calcium(II) release in TRIS buffer with a slight pH increase and improved mineral deposition that may have potential to improve osseointegration of hybrid glass scaffolds. Via the integration of bioactive glass microparticles in hybrid glass scaffolds, we can generate mechanically stable, bioactive implants that are promising for bone regeneration. 

## Figures and Tables

**Table 1:** Silica sol formulations used for fabrication of hybrid glasses (mol%).

Sol Formulation	TEOS	Calcium(II)	TEP
0C10P	90	0	10
2.5C10P	87.5	2.5	10
5C10P	85	5	10
10C10P	80	10	10

**Table 2:** Three-stage heating protocol.

Step	
1	heating from room temperature to 60 °C, dwell at 60 °C for 96 h
2	heating from 60 °C to 90 °C, dwell at 90 °C for 24 h
3	heating from 90 °C to 130 °C, dwell at 130 °C for 48 h

**Table 3:** Relative release of Calcium(II)/d from 10C10P, 5C10P, 5C10P and 1C10P scaffolds depending on the incubation time. 100% Calcium(II) were assumed to be equal to the cumulative release found in TRIS buffer after 84 days. Formulation names: 0 to 10 C: 0 to 10% Calcium(II) from addition of CaCl_2_, 10P: addition of 10% TEP.

	Ca(II) Release, [%/d]			
Incubation Time, [d]	10C10P	5C10P	2.5C10P	1C10P
0–1	98.37 ± 3.88	85.87 ± 3.89	72.36 ± 1.61	74.02 ± 3.47
1–3	0.09 ± 0.62	1.58 ± 1.59	4.65 ± 1.93	5.73 ± 0.45
3–7	0.30 ± 0.04	1.43 ± 0.18	2.09 ± 0.29	2.32 ± 0.05
7–14	0.04 ± 0.02	0.45 ± 0.09	0.73 ± 0.08	0.37 ± 0.05
14–21	0.00	0.13 ± 0.05	0.24 ± 0.09	0.36 ± 0.14
21–28	0.00	0.09 ± 0.04	0.15 ± 0.04	0.02 ± 0.04

**Table 4:** (**A**) Absolute release of Calcium(II)/d in 5 mL of incubation solution from 7 mg of BG-MP, 0C10P + BG-MP (7 mg), 2.5C10P + BG‑MP (14 mg) and (17 mg) scaffolds and 2.5C10P + BG-MP (14 mg) and (17 mg) scaffolds with subtracted 2.5C10P depending on the incubation time. (**B**) Average absolute calcium ions amount released in 5 mL of incubation solution per day. * For the calculation, the release after the first week of scaffold incubation was considered. Formulation names: 0 to 10 C: 0 to 10% Calcium(II) from addition of CaCl_2_, + BG MPs: addition of Vitryxx bioactive glass microparticles, 10P: addition of 10% TEP.

A	Calcium(II) Release [µg/5mL/d]					
time, [d]	7 mg BG-MP	0C10P + 7 mg BG-MP	2.5C10P + 14 mg BG‑MP	2.5C10P + 17 mg BG-MP	(2.5C10P + 14 mg BG-MP)-2.5C10P	(2.5C10P + 17 mg BG-MP)-2.5C10P
0–1	459.4 ± 110.1	11.9 ± 6.2	1003.1 ± 35.6	1023.1 ± 37.3	81.1 ± 11.6	77.5 ± 8.5
1–3	74.3 ± 36.5	14.3 ± 1.8	220.7 ± 14.6	186.0 ± 19.6	78.5 ± 43.2	55.5 ± 24.0
3–7	2.8 ± 1.0	14.6 ± 2.50	84.8 ± 21.7	103.7 ± 7.4	53.9 ± 24.3	72.8 ± 6.7
7–14	2.0 ± 1.3	10.7 ± 1.2	56.8 ± 2.4	71.9 ± 5.1	46.1 ± 2.1	61.1 ± 4.6
14–21	0.9 ± 0.9	9.3 ± 1.1	48.1 ± 1.0	54.7 ± 3.6	44.5 ± 1.6	51.1 ± 3.8
21–28	0.3 ± 0.5	10.4 ± 1.5	38.0 ± 2.7	48.3 ± 6.7	35.9 ± 3.1	46.2 ± 6.6
**B**	**Formulation**			**Average Calcium(II) Release Rate [µg/5mL/d]**		
	0C10P + 7 mg BG-MP			11.9		
	(2.5C10P + 14 mg BG-MP)–2.5C10P			42.1 *		
	(2.5C10P + 17 mg BG-MP)–2.5C10P			52.8 *		

## Appendix A

^1^H-NMR analysis showed stability of TEP against acid-catalyzed hydrolysis.

Recorded EDX spectra showed an absence of Phosphorous in fabricated hybrid glass scaffolds.

^1^H-NMR analysis showed TEP loss during the PCL-template leaching.

The adaptation of the scaffold curing protocol allowed to sufficiently incorporate the BG-microparticles.

Only minor scaffold degradation was shown during the 12 weeks degradation study with weight loss correlated to the content of water soluble calcium(II) chloride.

**Table A1:** Weight loss of selected hybrid glass scaffold formulations after incubation in TRIS-buffer solution for 84 d.

**Formulation**	**Weight Loss, wt%**
10C10P	26.9 ± 0.3%
5C10P	18.4 ± 1.3%
2.5C10P	14.4 ± 0.5%
0C10P	12.1 ± 0.3%
0C10P + BG-MP	11.3 ± 0.5%

Scaffold preincubation in cell culture medium resulted in HCA layer precipitation on the materials surface, similar to incubation in SBF.
